# Stable T-bet^+^GATA-3^+^ Th1/Th2 Hybrid Cells Arise *In Vivo*, Can Develop Directly from Naive Precursors, and Limit Immunopathologic Inflammation

**DOI:** 10.1371/journal.pbio.1001633

**Published:** 2013-08-20

**Authors:** Michael Peine, Sebastian Rausch, Caroline Helmstetter, Anja Fröhlich, Ahmed N. Hegazy, Anja A. Kühl, Christoph G. Grevelding, Thomas Höfer, Susanne Hartmann, Max Löhning

**Affiliations:** 1 Experimental Immunology, Department of Rheumatology and Clinical Immunology, Charité–University Medicine Berlin, Berlin, Germany; 2 German Rheumatism Research Center (DRFZ), a Leibniz Institute, Berlin, Germany; 3 Institute of Immunology, Department of Veterinary Medicine, Freie Universität Berlin, Berlin, Germany; 4 Department of Gastroenterology, Hepatology and Endocrinology, Charité, Berlin, Germany; 5 Department of Pathology and Research Center ImmunoSciences (RCIS), and Department of Gastroenterology, Infectiology, and Rheumatology, Charité, Berlin, Germany; 6 Institute of Parasitology, Justus-Liebig-University Giessen, Giessen, Germany; 7 Division of Theoretical Systems Biology, German Cancer Research Center (DKFZ), Heidelberg, Germany; 8 Bioquant Center, University of Heidelberg, Heidelberg, Germany; University of Pennsylvania, United States of America

## Abstract

Differentiated T helper (Th) cell lineages are thought to emerge from alternative cell fate decisions. However, recent studies indicated that differentiated Th cells can adopt mixed phenotypes during secondary immunological challenges. Here we show that natural primary immune responses against parasites generate bifunctional Th1 and Th2 hybrid cells that co-express the lineage-specifying transcription factors T-bet and GATA-3 and co-produce Th1 and Th2 cytokines. The integration of Th1-promoting interferon (IFN)-γ and interleukin (IL)-12 signals together with Th2-favoring IL-4 signals commits naive Th cells directly and homogeneously to the hybrid Th1/2 phenotype. Specifically, IFN-γ signals are essential for T-bet^+^GATA-3^+^ cells to develop *in vitro* and *in vivo* by breaking the dominance of IL-4 over IL-12 signals. The hybrid Th1/2 phenotype is stably maintained in memory cells *in vivo* for months. It resists reprogramming into classic Th1 or Th2 cells by Th1- or Th2-promoting stimuli, which rather induce quantitative modulations of the combined Th1 and Th2 programs without abolishing either. The hybrid phenotype is associated with intermediate manifestations of both Th1 and Th2 cell properties. Consistently, hybrid Th1/2 cells support inflammatory type-1 and type-2 immune responses but cause less immunopathology than Th1 and Th2 cells, respectively. Thus, we propose the self-limitation of effector T cells based on the stable cell-intrinsic balance of two opposing differentiation programs as a novel concept of how the immune system can prevent excessive inflammation.

## Abstract 2

The stable lineage commitment of naïve T helper cells to a hybrid Th1/2 phenotype reveals the cell-intrinsic reconciliation of two opposing T cell differentiation programs and provides a self-limiting mechanism to dampen immunopathology.

## Author Summary

T helper (Th) cells, a subgroup of white blood cells important in the immune system, can differentiate into diverse lineages, for example Th1 and Th2, whose effector mechanisms target different types of pathogens but cause problems if not properly regulated. Lineage commitment is driven by cytokine signals that control the expression of distinct lineage-specifying “master regulator” transcription factor molecules. Lineage commitment is thought to reflect alternative cell-fate decisions because the initiated differentiation programs have self-amplifying and mutually repressive features. Here we show that the Th1 and Th2 differentiation programs are more compatible with each other than previously thought. Individual naive T cells can simultaneously integrate Th1- and Th2-polarizing signals and develop into hybrid Th1/2 cells that stably co-express both the Th1 master regulator T-bet and the Th2 master regulator GATA-3. We find that hybrid Th1/2 cells arise naturally during parasite infections and that the two opposing differentiation programs can stably co-exist in resting memory Th1/2 cells for periods of months. Th1- or Th2-polarizing stimuli induced quantitative modulations in the hybrid state but did not extinguish either program. The cell-intrinsic antagonism gives the hybrid Th1/2 cells properties that are quantitatively intermediate between those of Th1 and Th2 cells. Thus, in typical Th1 and Th2 immune responses, hybrid Th1/2 cells cause less immunopathology than their classic Th1 or Th2 counterparts, demonstrating a cell-intrinsic self-limiting mechanism that can prevent excessive inflammation.

## Introduction

Upon antigen stimulation, polarizing cytokine signals initiate select differentiation programs in naive CD4^+^ T cells, resulting in the commitment to Th cell lineages with distinct functions [1]. Th1 cell differentiation is induced by IFN-γ [2],[3] and IL-12 [4],[5] signaling via STAT1 and STAT4, respectively, whereas Th2 cell differentiation is driven by IL-4 [6],[7] signaling via STAT6 [8].

The key transcription factor T-bet governs Th1 cell differentiation, which is associated with the acquisition of IFN-γ production [9] while the key transcription factor GATA-3 directs Th2 cell differentiation, resulting in the competence to produce IL-4, IL-13, and IL-5 [10],[11]. While all cells in a population of differentiated Th cells express their respective lineage-specifying transcription factor [12]–[15], the expression of cytokines during a given stimulation of such populations is heterogeneous [16],[17]. This points to a probabilistic element in acute cytokine production even though the differentiated population is in principle homogeneously competent to express its signature cytokine [18]–[22].

Given the distinct expression patterns of key transcription factors and cytokines in the Th cell differentiation lineages together with multiple mechanisms of positive feedback [2],[23],[24] and reciprocal inhibition [25]–[27], the Th1 and Th2 differentiation programs were assumed to be mutually exclusive [28]. However, already soon after the first description of Th1 and Th2 cells [29], reports emerged of both human and murine T cell clones that co-expressed Th1 and Th2 cytokines such as IFN-γ and IL-4 [30],[31]. While the differentiation history of these cells was unclear, these findings were reconciled with the concept of Th1/Th2 dichotomy by the idea that upon activation naive cells may pass through an uncommitted and pluripotent state, but that ongoing stimulation would finally lead to the stable classic Th1 and Th2 lineages [32]–[34].

Recently, the plasticity of Th cell lineages and its functional relevance have been intensively discussed [35]–[37]. The observation of several hybrid differentiation phenotypes revealed formerly unappreciated flexibility of the classic Th cell lineages [12],[38]–[44]. However, the concerted stable commitment of naive Th cells to two mutually repressive differentiation programs, such as the Th1 and the Th2 programs, has not been described yet. In particular, whether T-bet^+^GATA-3^+^ cells develop in natural immune responses has been unclear. Still, *in vivo* responses to certain parasites, while exhibiting a strong Th2 bias [45], may also generate Th cells that can co-express Th1 and Th2 cytokines [46]. This prompted us to investigate Th cell differentiation during parasite infections.

Here we show that in natural immune responses against parasites, hybrid Th1/2 cells developed in parallel to classic Th2 cells. Highly purified naive Th cells could simultaneously and homogeneously commit to the Th1 and Th2 differentiation programs by the integration of IFN-γ, IL-12, and IL-4 signals. The resulting hybrid Th1/2 cells stably co-expressed T-bet and GATA-3 *in vivo* for months and upon challenge co-produced Th1 and Th2 cytokines. The stable balance of the two antagonistic differentiation programs in hybrid Th1/2 cells was associated with intermediate manifestation of Th1 and Th2 cell properties and translated into attenuated immunopathology in murine models of type-1 and type-2 inflammation. These findings suggest a novel role of a hybrid differentiation phenotype for the limitation of excessive inflammatory immune responses.

## Results

### Hybrid Th1/2 Cells Develop *In Vivo* in Response to Parasite Infections

**Figure 1:**
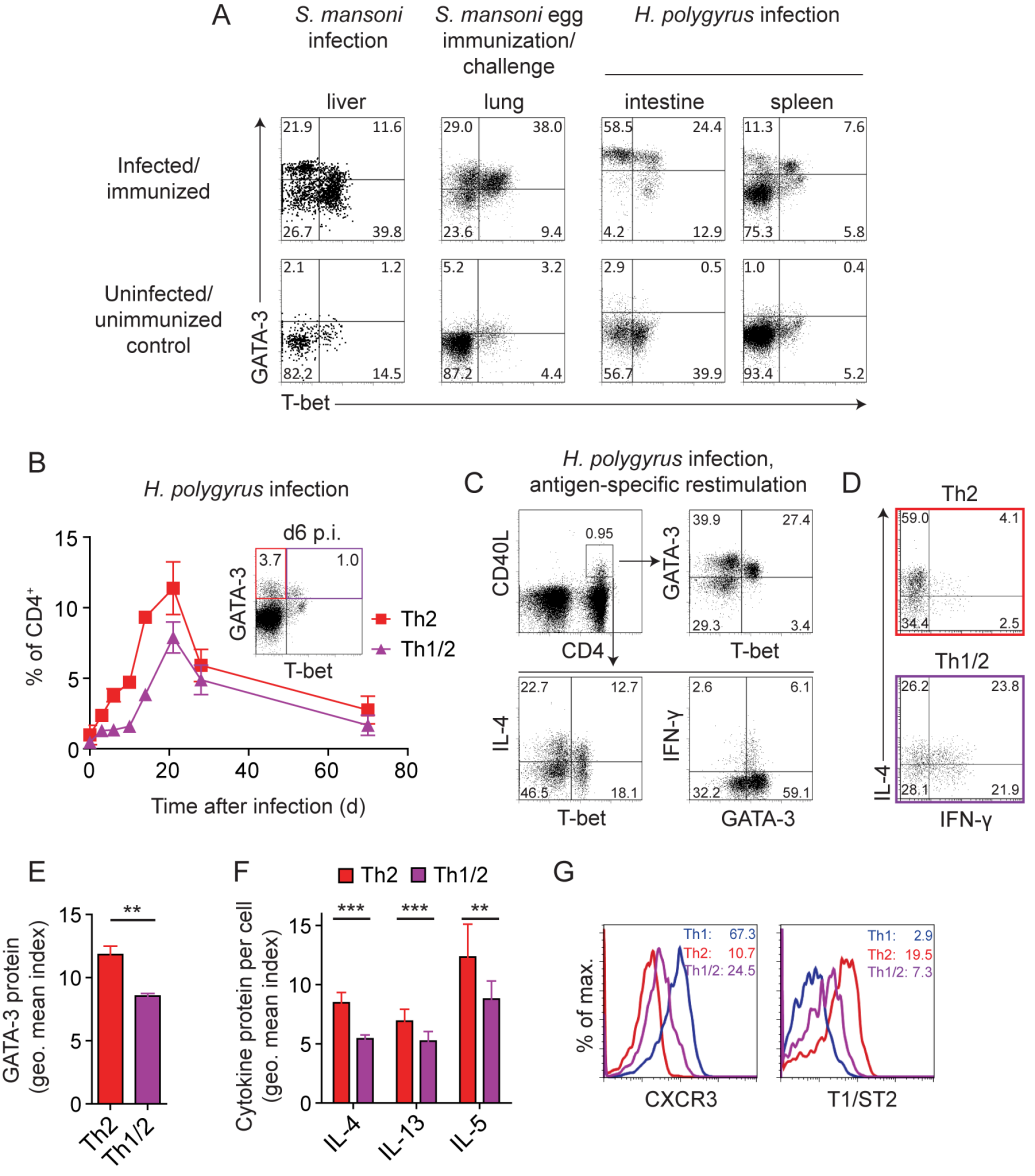
Hybrid Th1/2 cells develop *in vivo* in response to parasite infections. (A) WT C57BL/6 or Balb/c mice were infected or immunized/challenged with the indicated pathogens. T-bet and GATA-3 expression by CD4^+^ T cells from indicated organs was analyzed *ex vivo* without restimulation. Numbers indicate frequencies. Data are representative of three *H. polygyrus* and three *S. mansoni* experiments with *n* = 3–4 mice each. (B) WT C57BL/6 mice were infected with *H. polygyrus*. T-bet and GATA-3 expression in CD4^+^ splenocytes was analyzed in a kinetic fashion. Mean frequencies ± SD of T-bet^−^GATA-3^+^ (Th2) and T-bet^+^GATA-3^+^ (Th1/2) cells are shown (*n* = 3 mice/time point). Inserted dot plot shows a typical staining on d 6. Data are representative of two independent experiments. (C) Spleen and mLN cells from WT C57BL/6 mice infected with *H. polygyrus* were restimulated with adult worm lysate at the peak of infection on d 21. Antigen-specific CD4^+^ T cells as identified by CD40L expression were analyzed for transcription factor and cytokine expression. Numbers indicate frequencies. Data are representative of three independent experiments. (D–F) WT C57BL/6 mice were infected with *H. polygyrus*. CD4^+^ splenocytes were analyzed at the peak of infection on d 21. Th2 cells were gated as CD4^+^T-bet^−^GATA-3^+^ and Th1/2 cells as CD4^+^T-bet^+^GATA-3^+^ as shown in (B). (D) Cytokine expression was analyzed upon PMA/ionomycin restimulation (*n* = 6 mice). Numbers indicate frequencies. Data are representative of two independent experiments. (E) Geometric mean indices ± SD of GATA-3 protein staining are shown (*n* = 3 mice). Data are representative of three independent experiments. (F) Spleen cells were restimulated as in (D). Geometric mean indices ± SD of cytokine expression calculated as the geometric mean of cytokine^+^ cells divided by the geometric mean of cytokine^−^ cells are shown (*n* = 6 mice). Data are pooled from two independent experiments. (G) Expression of CXCR3 and T1/ST2 by CD4^+^ T cells was analyzed on day 14 of infection. Th2 cells were gated as CD4^+^T-bet^−^GATA-3^+^ and Th1/2 cells as CD4^+^T-bet^+^GATA-3^+^ as shown in (B). As a Th1 reference, the T-bet^+^GATA-3^−^ population was gated as in the lower right quadrant of the inset in (B). Numbers indicate geometric means. Data are representative of two independent experiments.

To determine whether T-bet^+^GATA-3^+^ cells develop in unmanipulated immune reactions, we used different parasite infections that elicit Th2-biased immune responses [45]. Infection with *Schistosoma mansoni* cercariae induced bona fide Th2 cells expressing GATA-3 as well as a substantial cell population co-expressing GATA-3 and T-bet at the single-cell level as cell-autonomous hybrid cells (Figure 1A). *S. mansoni* infections elicit an early Th1 and a later, egg-dependent, Th2 response [47], leaving the possibility that the T-bet^+^GATA-3^+^ cells we observed were reprogrammed Th1 cells. To address whether cells can co-express T-bet and GATA-3 during a strongly Th2-biased response [48],[49], we directly immunized and challenged mice with *S. mansoni* eggs, inducing type-II granulomas in the lung [50]. In addition, to generate a primary Th2-polarized response, we infected mice with *Heligmosomoides polygyrus*. Similar to the response induced by *S. mansoni* cercariae, we found GATA-3^+^ cells together with considerable fractions of T-bet^+^GATA-3^+^ cells in the lungs of mice challenged with *S. mansoni* eggs as well as in the intestines, mesenteric LN (mLN), and spleens of *H. polygyrus*–infected mice (Figure 1A and unpublished data). Moreover, following the emergence of GATA-3^+^ and T-bet^+^GATA-3^+^ cells during *H. polygyrus* infection in a time-resolved fashion, we detected both subsets earliest on day 6 after infection. The subsequent expansion and contraction of both compartments followed the same kinetics (Figure 1B). Taken together, these data indicate that hybrid Th1/2 cells co-expressing T-bet and GATA-3 at the single-cell level emerged naturally during primary and secondary type-2 immune responses. Of note, hybrid Th1/2 cells developed in both C57BL/6 and Balb/c mice in response to both parasites (Figure 1A and unpublished data).

GATA-3^+^ cells in general and Th2 cytokine-producing cells were largely absent from control mice (Figure 1A and unpublished data). Yet, to directly assess the antigen specificity of *in vivo*–generated T-bet^−^GATA-3^+^ and T-bet^+^GATA-3^+^ cells, we restimulated cells from the spleens and mLN of *H. polygyrus*–infected mice with adult worm lysate and identified antigen-specific T cells by CD40L expression [51]. *H. polygyrus*–specific cells contained both T-bet^−^GATA-3^+^ and T-bet^+^GATA-3^+^ cells (Figure 1C, upper row). Effector cells producing IL-4 were present in both the T-bet^−^ and T-bet^+^ compartment (Figure 1C, lower row). Conversely, IFN-γ producers were found in the CD40L^+^GATA-3^+^ compartment. In particular, hybrid T-bet^+^GATA-3^+^ Th1/2 cells coproduced IFN-γ and IL-4, whereas T-bet^−^GATA-3^+^ Th2 cells primarily produced IL-4 but not IFN-γ (Figure 1D). At a quantitative level, the amount per cell of both GATA-3 protein (Figure 1C and E) and IL-4, IL-13, and IL-5 proteins (Figure 1C and F) was reduced in T-bet^+^GATA-3^+^ cells compared with their T-bet^−^ counterparts.

Of note, hybrid Th1/2 cells formed a homogeneous population that co-expressed the lineage-specifying transcription factors T-bet and GATA-3 while upon restimulation IFN-γ and IL-4 were produced by fractions of this population. This probabilistic cytokine expression behavior of the homogeneously differentiated hybrid Th cell population mirrored the stochastic cytokine expression of classic Th1 and Th2 cell populations [18]–[22] where only a fraction of the differentiated cells produces IFN-γ or IL-4, respectively, in a given restimulation [16],[17]. Consistent with earlier reports [46],[52], IFN-γ/IL-4 co-expression occurred at frequencies expected for stochastically independent events, indicating a superimposition of the Th1 and Th2 transcriptional programs in the individual hybrid cells. In contrast, hybrid Th1/2 cells homogeneously expressed intermediate levels of the Th1-associated chemokine receptor CXCR3 [53],[54] and the Th2-associated IL-33 receptor T1/ST2 [46],[55] when compared with their classic T-bet^−^GATA-3^+^ Th2 counterparts and a reference population of bulk T-bet^+^GATA-3^−^ effector/memory Th1 cells (Figure 1G).

In summary, our data show that hybrid Th1/2 cells developed in several natural immune responses against different parasites alongside with classic Th2 cells. Hybrid Th1/2 cells exhibited a bifunctional type-1 and type-2 cytokine response and displayed Th2 features at an intermediate magnitude.

### Simultaneous Commitment of Naive T Helper Cells to the Th1 and Th2 Differentiation Programs

The parallel emergence of hybrid T-bet^+^GATA-3^+^ Th1/2 cells together with classic T-bet^−^GATA-3^+^ Th2 cells during *H. polygyrus* infection suggested that the hybrid cells may directly differentiate from naive precursors since they accompanied their T-bet^−^ Th2 counterparts at any time point analyzed. To formally address whether simultaneous differentiation of naive Th cells along the Th1 and Th2 pathways is possible, we applied a mixed cytokine environment, containing both Th1- and Th2-polarizing stimuli. We highly sort-purified naive T cells and cultured them with IFN-γ and IL-12 to induce Th1 differentiation, IL-4 to induce Th2 differentiation, or a combination of IFN-γ, IL-12, and IL-4 to test the effect of a promiscuous cytokine environment.

**Figure 2:**
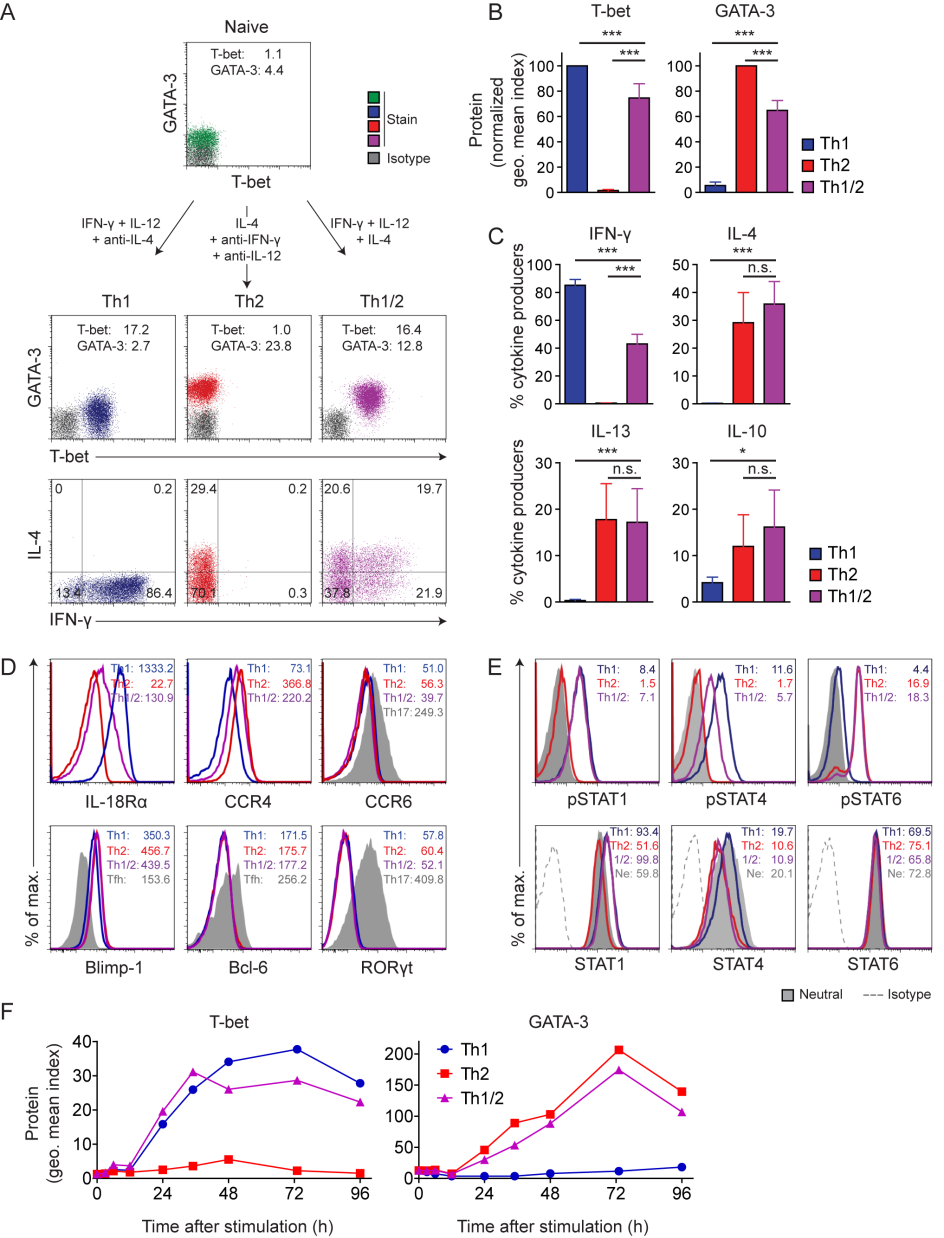
Simultaneous commitment of naive T cells to the Th1 and Th2 differentiation programs. (A–E) FACS-sorted naive CD4^+^CD62L^hi^CD44^lo^CD25^−^CXCR3^−^Gr-1^−^ LCMV-TCR^tg^ Th cells were activated with APCs and GP_61–80_ peptide in the presence of the indicated cytokines and cytokine-blocking antibodies. (A) T-bet and GATA-3 expression is shown in naive cells (top row) or on d 5 of culture (middle row). Geometric mean indices are indicated. Cytokine expression upon PMA/ionomycin restimulation is shown on d 5 of culture (lower row). Numbers indicate frequencies. Data are representative of five independent experiments. (B) Geometric mean indices ± SD of T-bet and GATA-3 stainings versus isotype control stainings on d 5 of culture are depicted as normalization to Th1 cells for T-bet or to Th2 cells for GATA-3. Data are pooled from five independent experiments. (C) Cytokine expression was analyzed after PMA/ionomycin restimulation on d 5 of culture. Mean frequencies ± SD of cytokine^+^ cells are shown. Data are pooled from five independent experiments. (D) Expression of the indicated cytokine and chemokine receptors (upper row) and transcription factors (lower row) was analyzed on d 5 of culture. Tfh cells were obtained from spleens of WT C57BL/6 mice and identified as CD4^+^CXCR5^+^PD-1^+^ cells. Numbers indicate geometric means. Data are representative of two independent experiments. (E) On d 2 of culture, levels of phosphorylated (upper row) and total (lower row) STAT proteins were determined. Numbers indicate geometric mean indices. Data are representative of two independent experiments. (F) Naive Th cells from WT Balb/c mice were cultured with anti-CD3/anti-CD28 in the presence of polarizing cytokines and cyokine-blocking antibodies to induce Th1, Th2, and hybrid Th1/2 cells. T-bet and GATA-3 expression was analyzed in a kinetic fashion and plotted as geometric mean indices. Data are representative of three independent experiments.

As expected, the first two conditions gave rise to the well-established dichotomy of Th1 or Th2 cells with differential expression of T-bet or GATA-3, respectively, and production of IFN-γ or IL-4 and IL-13, respectively (Figure 2A–C). Cells cultured with the combination of IFN-γ, IL-12, and IL-4 displayed a hybrid Th1/2 phenotype, co-expressing T-bet and GATA-3 homogeneously and co-producing IFN-γ together with IL-4 and IL-13 (Figure 2A and unpublished data). As found for the *in vivo*–differentiated hybrid cells and consistent with earlier reports [46],[52], IFN-γ was co-expressed with IL-4 or IL-13 at frequencies expected for stochastically independent events.

All hybrid Th1/2 cells stained positive for both T-bet and GATA-3 protein, and the amount per cell of either key transcription factor was in between that of the classic lineages (Figure 2B). The frequency of IFN-γ producers was about halved in hybrid Th1/2 cells compared to Th1 cells, while the frequencies of IL-4, IL-13, and IL-10 producers were similar to those in Th2 cells (Figure 2C). Beyond the co-expression of T-bet and GATA-3 and the co-production of IFN-γ and IL-4, the hybrid cells exhibited intermediate expression levels of the Th1-associated cytokine receptor IL-18Rα [56] as well as of the Th2-associated chemokine receptor CCR4 [53] but they failed to express the Th17-associated chemokine receptor CCR6 (Figure 2D). Similar to the Th1 and Th2 effector cell lineages, hybrid Th1/2 cells expressed the transcription factor Blimp-1. However, hybrid Th1/2 cells did not express defining transcription factors of other lineages, such as the T follicular helper (Tfh)- and Th17 cell-specifying transcription factors Bcl-6 and RORγt, respectively (Figure 2D), or the key transcription factor of regulatory T cells, Foxp3 (unpublished data).

Next we asked whether quantitative modulations of Th1 and Th2 cell features in hybrid Th1/2 cells resulted from intermediate signal strength exerted by adverse polarizing cytokines. Analyzing the phosphorylation of STAT proteins in differentiating cells, we found that the key STAT signaling pathways downstream of IFN-γ, IL-12, and IL-4 were all active in the entire cell population during the induction of hybrid Th1/2 cells (Figure 2E). However, in quantitative terms, levels of phosphorylated STAT4 but not phosphorylated STAT1 were reduced in hybrid Th1/2 cells compared with Th1 cells, correlating with reduced expression of total STAT4 but not STAT1 protein. Expression and phosphorylation of STAT6 was similar in hybrid Th1/2 and Th2 cells.

To address whether naive cells exposed to IFN-γ, IL-12, and IL-4 would first adopt either the Th1 or the Th2 differentiation program and get reprogrammed to the hybrid phenotype at a later stage, we quantified T-bet and GATA-3 proteins in a detailed kinetic fashion (0 h, 3 h, 6 h, 12 h, 24 h, 35 h, 48 h, 73 h, 95 h) during primary differentiation. We found that the time point of homogeneous induction of the lineage-specifying transcription factors and their expression dynamics were identical in hybrid Th1/2 cells and their classic Th1 and Th2 counterparts (Figure 2F). Thus, individual naive Th cells can directly differentiate into hybrid Th1/2 cells in a primary response, co-expressing key transcription factors and effector molecules of both the Th1 and the Th2 cell lineages.

### IFN-γ– and IL-4–Producing Th Cells Are Distributed over the Entire Spectrum of T-bet and GATA-3 Expression Levels

**Figure 3:**
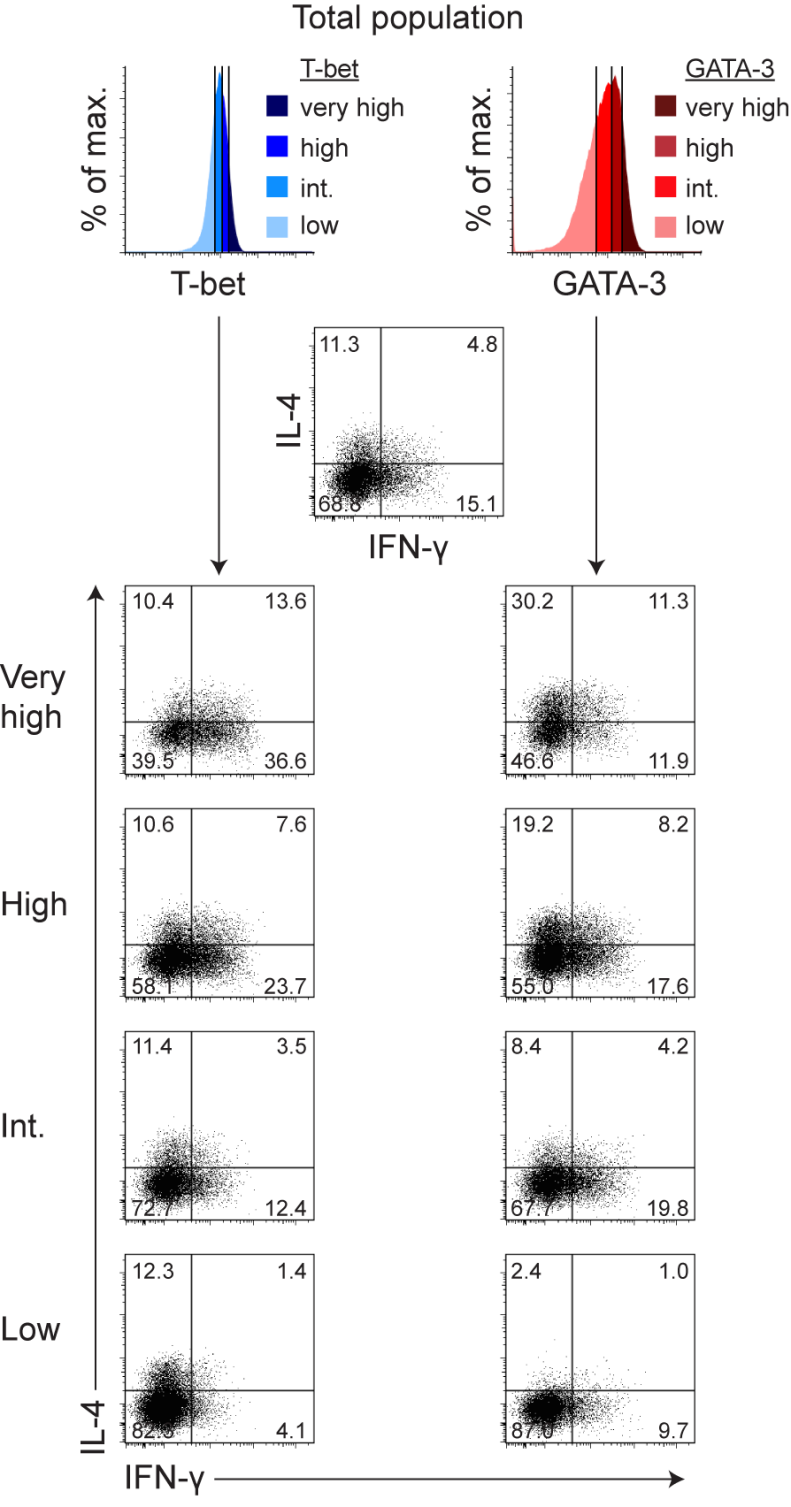
IFN-γ– and IL-4–producing hybrid Th1/2 cells are distributed over the entire spectrum of T-bet and GATA-3 expression levels. FACS-sorted naive LCMV-TCR^tg^ CD4^+^ T cells were cultured for 5 d with APCs and GP_64–80_ peptide to generate hybrid Th1/2 cells. Transcription factor and cytokine co-expression was analyzed after PMA/ionomycin restimulation. Dot plots show the co-expression of IFN-γ and IL-4 within fractions expressing distinct levels of T-bet (left column) or GATA-3 (right column) that were gated according to the colored histograms on top. As very high fraction, the 10% of cells expressing the highest levels of the respective transcription factor were analyzed. The other fractions each contained approximately 30% of the total population. IFN-γ and IL-4 production in the whole population is depicted in the top center dot plot. Numbers indicate frequencies. Data are representative of two independent experiments.

Hybrid Th1/2 cells homogeneously co-expressed T-bet and GATA-3, whereas upon restimulation, as expected from the probabilistic cytokine expression behavior of classic Th1 and Th2 cells, only subpopulations stained positive for IFN-γ, IL-4, or IL-13 (Figure 2A). It has been shown that Th1 and Th2 cells purified according to the secretion of a certain cytokine do not reexpress this cytokine in a deterministic manner in subsequent stimulations. Instead, sorted cytokine-producing and -nonproducing fractions each segregated again into cytokine producers and nonproducers, consistent with stochastic events underlying cytokine gene expression [18]–[22]. These studies showed that also those Th cells from a differentiated population that do not produce lineage-specific cytokines in a given stimulation still have the general capacity to do so and can express these cytokines in a subsequent challenge, indicating that they are bona fide Th1 or Th2 cells. Thus, the expression of lineage-specifying “master” transcription factors such as T-bet and GATA-3, which are upstream regulators of the Th1 and Th2 differentiation programs including the cytokine genes, have become recognized as the major criterion for lineage identity and commitment [35],[36] since they are constitutively expressed by all cells of a lineage [12],[13]. Hence, the probabilistic cytokine production behavior of hybrid Th1/2 cells, all of which expressed both T-bet and GATA-3, matched that of classic Th1 and Th2 cells. The hybrid Th1/2 population contained cells producing only IFN-γ, only IL-4, or co-producing both (Figure 2A), consistent with the stochastically independent production of different cytokines by Th cells [46],[52]. However, to analyze whether some of the hybrid cells were more Th1-like and others more Th2-like, we performed a quantitative co-expression analysis of lineage-defining transcription factors and cytokines (Figure 3). Even the 10% of the cells expressing the highest levels of either lineage-specifying transcription factor contained both IFN-γ– and IL-4–producing cells, indicating that they did not represent “contaminating” Th1 or Th2 cells. T-bet and GATA-3 expression levels correlated with the probability of cytokine expression, but IFN-γ and IL-4 producers were distributed over the entire population, and high T-bet and GATA-3 expression did not predict cytokine production of individual cells in a deterministic manner, supporting the concept of probabilistic cytokine gene expression [18]–[22]. Interestingly, high expression of either transcription factor increased the likelihood of both IFN-γ and IL-4 production, possibly due to a somewhat higher activation state of these cells.

Taken together, the Th1/2 population represented homogeneously differentiated cell-autonomous hybrid cells. An elevated expression level of either master transcription factor was not associated with a classic Th1 or Th2 cytokine pattern and thus did not indicate an exclusive lineage choice.

### Induction of T-bet and IFN-γ in the Presence of IL-4 Critically Depends on IFN-γ Signaling

**Figure 4:**
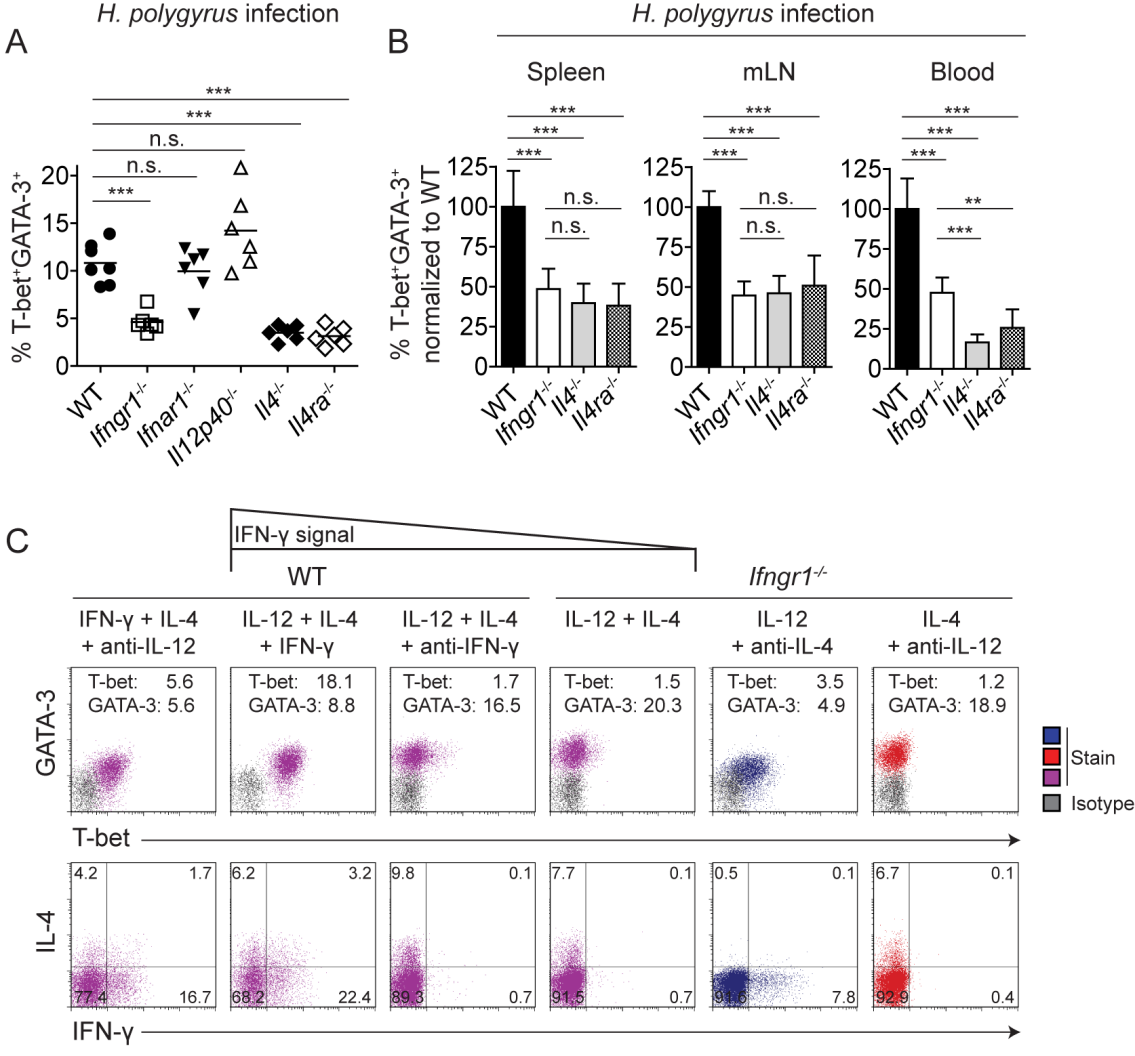
Induction of T-bet and IFN-γ in the presence of IL-4 critically depends on IFN-γ signaling. (A) WT C57BL/6 mice or the indicated gene-deficient mice were infected with *H. polygyrus*. On d 20 of infection, CD4^+^ T cells from spleens were analyzed for T-bet and GATA-3 expression (*n* = 6–7 mice/group). The frequencies of T-bet^+^GATA-3^+^ hybrid Th1/2 cells among activated CD4^+^CD44^+^ T cells are shown. (B) The frequencies of T-bet^+^GATA-3^+^ hybrid Th1/2 cells among activated CD4^+^CD44^+^ T cells as shown in (A) in the indicated organs were normalized to the mean of the wild-type group. Data are representative of two independent experiments. (C) FACS-sorted naive CD4^+^CD62L^hi^CD44^lo^CD25^−^CXCR3^−^Gr-1^−^ Th cells from C57BL/6 or *Ifngr^−/−^* mice were activated with anti-CD3/anti-CD28 in the presence of the indicated cytokines and cytokine-blocking antibodies. T-bet and GATA-3 expression was analyzed on d 5 (upper row). Inserted numbers indicate geometric mean indices. Cytokine expression was analyzed upon PMA/ionomycin restimulation on d 5 (lower row). Numbers indicate frequencies. Data are representative of two independent experiments.

To identify the signals that induced hybrid Th1/2 cells *in vivo*, we infected different cytokine- or cytokine receptor-deficient mice with *H. polygyrus*. *H. polygyrus*–infected *Ifngr1^−/−^*, *Il4^−/−^*, or *Il4ra^−/−^* mice exhibited a strong reduction in the frequency of T-bet^+^GATA-3^+^ hybrid Th1/2 cells compared with wild-type mice (Figure 4A). This defect was observed in the spleens as well as in the mLN and the peripheral blood (Figure 4B). In contrast, we detected unaltered frequencies of hybrid Th1/2 cells in *Il12p40^−/−^* and *Ifnar1^−/−^* mice (Figure 4A). In the blood but not in spleens and mLN, the absence of IL-4 signals affected the development of hybrid Th1/2 cells even more strongly than the absence of IFN-γ signals (Figure 4B). Thus, IFN-γ and IL-4 but not IL-12 or type I interferons were required to generate hybrid Th1/2 cells during *H. polygyrus* infection, and this requirement was not only evident at the peak of the response (d 20) but already at an earlier stage (d 14, unpublished data).

Although IL-12 often critically contributes to Th1 cell differentiation [4],[5], multiple pathways to induce T-bet [13] can render IL-12 dispensable in many Th1 immune responses [57]–[59]. Therefore, we asked whether IFN-γ alone was sufficient to induce T-bet and IFN-γ in naive Th cells in the presence of adverse IL-4 signals. To rule out confounding IL-12 production by antigen-presenting cells (APCs), we stimulated purified naive Th cells with plate-bound anti-CD3 and anti-CD28 in the presence of IFN-γ and IL-4. Indeed, this treatment resulted in a cell population homogeneously co-expressing T-bet and GATA-3, albeit at a suboptimal magnitude, and co-producing IFN-γ and IL-4 (Figure 4C and Figure S1). The further addition of IL-12 markedly increased the expression of T-bet and the capacity for IFN-γ production. Of note, both the frequencies of cytokine producers and the expression levels of key transcription factors were generally lower with anti-CD3/anti-CD28 stimulation than with peptide-APC stimulation.

Given that IL-12–STAT4 signaling in differentiating hybrid Th1/2 cells was compromised due to the presence of simultaneous IL-4 signals (cf. Figure 2E), we asked whether parallel interference with IFN-γ signals would be critical for inducing the Th1 program in hybrid Th1/2 cells. When we added an IFN-γ–blocking antibody to cells cultured with IL-12 and IL-4, T-bet expression was almost completely abrogated, and IFN-γ production was totally extinguished (Figure 4C). Similarly, naive Th cells from *Ifngr1^−/−^* mice cultured with IL-12 and IL-4 exhibited a Th2-like phenotype, completely lacking T-bet and IFN-γ expression. By contrast, Th1 cells from *Ifngr1^−/−^* mice cultured with IL-12 alone at least showed elevated T-bet expression and contained a sizable fraction of IFN-γ producers.

Collectively, these data indicate that IFN-γ signals, acting directly on differentiating Th cells, could overcome the inhibitory effects of the IL-4–GATA-3 axis on T-bet induction and hence were critical for the development of hybrid Th1/2 cells.

### Hybrid Th1/2 Memory Cells Stably Maintain Their Key Transcription Factor and Cytokine Profile

**Figure 5:**
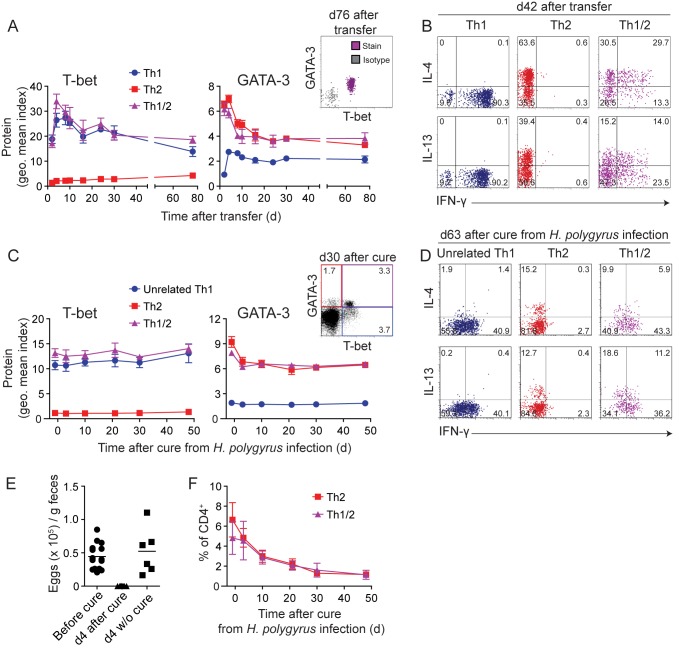
Hybrid Th1/2 memory cells stably maintain their key transcription factor and cytokine profile. (A–B) LCMV-TCR^tg^ Thy1.1^+^ Th1, Th2, or hybrid Th1/2 cells were generated *in vitro* for 2 wk and transferred into WT C57BL/6 mice (*n* = 3–8 mice/group). Data are representative of two independent experiments. (A) T-bet and GATA-3 expression in CD4^+^Thy1.1^+^ cells from peripheral blood was analyzed in a kinetic fashion and plotted as geometric mean indices ± SD. Dot plot depicts a representative staining on d 76 after transfer. (B) Splenocytes were restimulated with GP_64–80_ peptide on d 42 after transfer and cytokine production of CD4^+^Thy1.1^+^ donor cells was analyzed. Numbers indicate frequencies. (C–F) WT C57BL/6 mice were infected with *H. polygyrus* and cured with Pyrantel at the peak of infection on d 21 (*n* = 10). Data are representative of two independent experiments. (C) T-bet and GATA-3 expression was determined in CD4^+^ cells from peripheral blood in a kinetic fashion and plotted as geometric mean indices ± SD. Dot plot (right panel) depicts a representative staining on d 30 after cure. Numbers indicate frequencies. The blue, red, and violet frames in this inset also document the gating strategy for Th1, Th2, and hybrid Th1/2 cells, respectively, which are shown in panels (C), (D), and (F). (D) Cytokine expression upon restimulation with PMA/ionomycin was analyzed in CD4^+^ cells from spleens of *H. polygyrus*–infected mice on d 63 after cure. Numbers indicate frequencies. (E) Output of *H. polygyrus* eggs was determined one day before and 4 d after treatment with Pyrantel. (F) Frequencies ± SD of T-bet^−^GATA-3^+^ (Th2) and T-bet^+^GATA-3^+^ (hybrid Th1/2) CD4^+^ cells in the peripheral blood are depicted over time after cure from *H. polygyrus* infection.

Th1 and Th2 cells maintain enhanced levels of T-bet and GATA-3 protein, respectively, in the memory phase when the inducing cytokine environment is no longer present [12]. Considering the mechanisms of reciprocal inhibition of Th1 and Th2 differentiation programs, we asked whether hybrid Th1/2 cells represented a transitory state, dependent on a promiscuous cytokine environment during antigen stimulation, or whether the hybrid phenotype was similarly stable as that of Th1 and Th2 cells. To this end, we generated pure populations of Th1, Th2, and hybrid Th1/2 cells *in vitro* as shown by the uniform expression patterns of T-bet and GATA-3 (cf. Figure 2A), transferred them into wild-type mice, and tracked them in the recipients for several months. Throughout the observation period, hybrid Th1/2 memory cells homogeneously continued to co-express T-bet and GATA-3 (Figure 5A, right). The hybrid cells maintained the expression of T-bet at a similar level as in Th1 cells and of GATA-3 at a similar level as in Th2 cells (Figure 5A, left and center) and continued to co-express IFN-γ with IL-4 and IL-13 upon restimulation (Figure 5B), indicating the stable maintenance of the hybrid Th1/2 phenotype in the memory phase *in vivo*.

*H. polygyrus* infection is usually chronic. Therefore, to study the memory phase of *in vivo*–generated hybrid Th1/2 cells, we cured mice with an anti-helminth drug at the peak of infection. After expulsion of the parasites, *H. polygyrus*–induced T-bet^+^GATA-3^+^ cells maintained GATA-3 expression similar to their T-bet^−^ Th2 counterparts and constantly expressed T-bet at levels like the pool of T-bet^+^ Th1 effector/memory cells unrelated to the parasite infection (Figure 5C). They also contributed to the memory pool of cytokine producers and co-produced IFN-γ with IL-4 and IL-13 (Figure 5D). Of note, we verified the complete absence of egg output after cure for each mouse (Figure 5E). Hybrid Th1/2 cells in *H. polygyrus*–cured mice accounted for approximately half of the GATA-3^+^ memory pool (Figure 5F). Thus, bifunctional Th1/2 cells did not represent a transient effector stage but rather maintained their hybrid phenotype in the memory phase.

### The Hybrid Th1/2 Phenotype Can Be Quantitatively Modulated but Resists Full Reprogramming into Th1 or Th2 Cells

The Th1 and Th2 differentiation programs exhibit features of mutual inhibition. Therefore, one could speculate that either program in hybrid Th1/2 cells would be extinguished upon secondary unidirectional challenges. To address this possibility, we stimulated hybrid Th1/2 cells for a second round under either Th1-favoring conditions including type I and type II interferons [12] or Th2-polarizing conditions.

**Figure 6:**
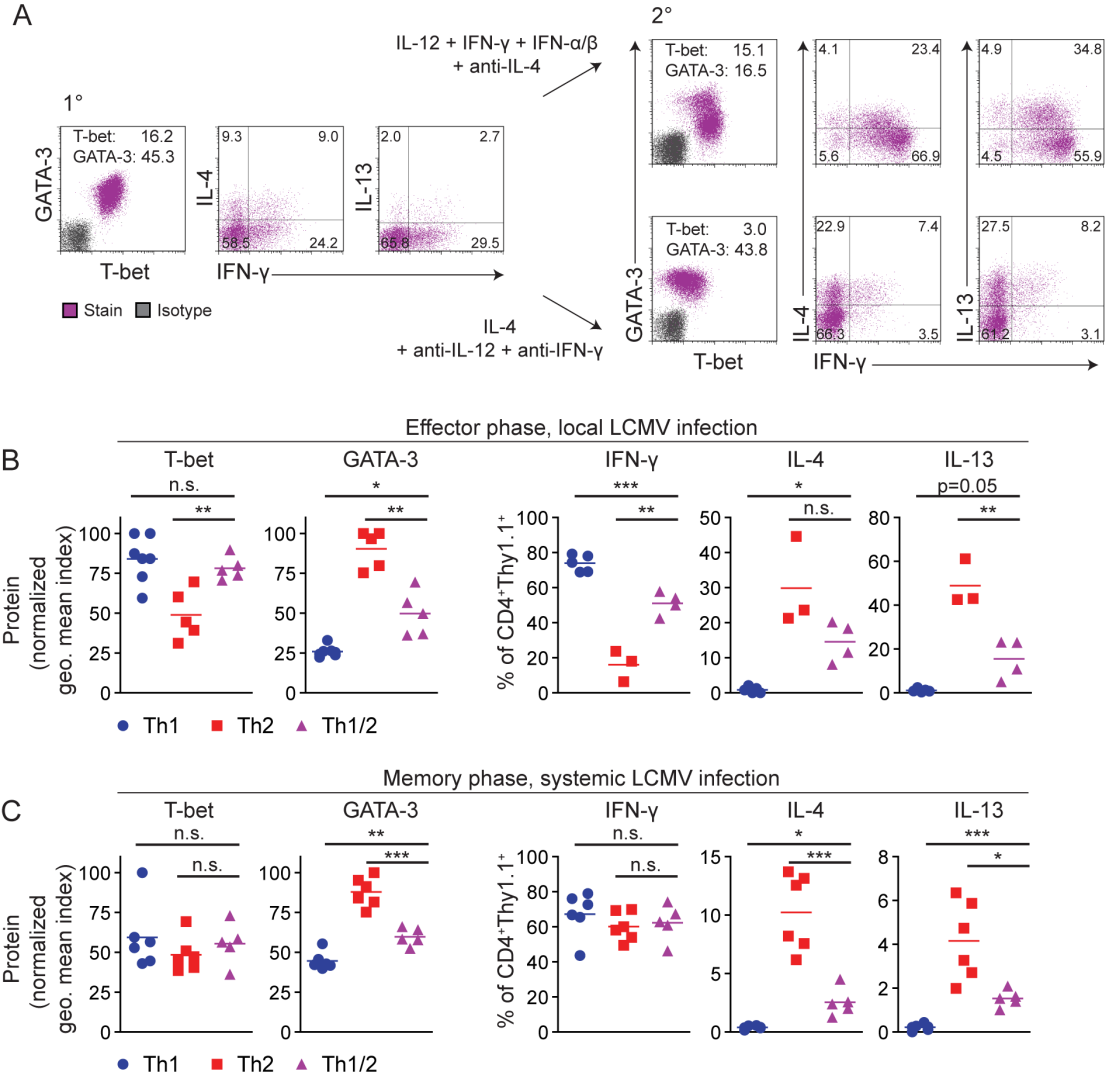
The hybrid Th1/2 phenotype can be quantitatively modulated but resists full reprogramming into Th1 or Th2 cells. FACS-sorted naive LCMV-TCR^tg^ CD4^+^Thy1.1^+^ T cells were cultured for 5 d with APCs and GP_64–80_ peptide to generate Th1, Th2, or hybrid Th1/2 cells. (A) Hybrid Th1/2 cells were analyzed on d 5 (left) and further stimulated for 5 d in the presence of either Th1-reprogramming conditions (upper row) or Th2-polarizing conditions (lower row). Geometric mean indices of T-bet and GATA-3 expression are indicated. Cytokine expression was analyzed after PMA/ionomycin restimulation. Numbers indicate frequencies. Data are representative of two independent experiments. (B) 5×10^6^ Th1, Th2, or hybrid Th1/2 cells were transferred into WT mice, followed by subcutaneous LCMV infection of all recipients in the footpad. CD4^+^Thy1.1^+^ donor cells were harvested from the feet during the effector phase on d 5–8 after infection. Normalized geometric mean indices of T-bet and GATA-3 expression without restimulation are shown (*n* = 5–7 mice/group). Data are pooled from two independent experiments. Frequencies of cytokine^+^ cells were analyzed after PMA/ionomycin restimulation (*n* = 3–5 mice/group). Data are representative of two independent experiments. (C) 1×10^6^ Th1, Th2, or hybrid Th1/2 cells were transferred into WT C57BL/6 mice, followed by intravenous LCMV infection of all recipients. Splenocytes were analyzed on d 71. Normalized geometric mean indices of T-bet and GATA-3 expression in CD4^+^Thy1.1^+^ cells without restimulation are shown for individual mice (left panels). Frequencies of cytokine^+^ cells among CD4^+^Thy1.1^+^ cells after PMA/ionomycin restimulation are depicted (*n* = 5–6 mice/group). Data are representative of two independent experiments.

Cells subjected to Th1-favoring conditions maintained high T-bet expression, while GATA-3 expression was reduced but not abrogated. The frequency of IFN-γ producers was drastically enhanced, but many of them co-expressed IL-4 and IL-13 (Figure 6A). Under Th2-polarizing conditions, high GATA-3 expression was maintained, and T-bet as well as IFN-γ expression was further reduced but not abrogated. The frequencies of IL-4 and IL-13 producers were enhanced, however these frequencies were similar to those after Th1-favoring challenge. Thus, while the main effect of secondary Th1 challenge was to overcome diminished IFN-γ production in hybrid Th1/2 cells, the main effect of secondary Th2 challenge was the more pronounced suppression but not extinction of IFN-γ and T-bet expression.

Infections with lymphocytic choriomeningitis virus (LCMV) provide potent stimuli for Th1-cell polarization [12]. Hence, to study the plasticity of hybrid Th1/2 cells versus classic Th1 and Th2 cells under strong Th1-favoring conditions *in vivo*, we transferred LCMV-specific Th1, Th2, and hybrid Th1/2 cells into mice, followed by a local LCMV infection into the footpad. At the peak of infection, we isolated cells directly from the challenged feet. We found that hybrid Th1/2 cells had acquired similar T-bet expression as Th1 cells, whereas the frequency of IFN-γ producers remained at an intermediate level between Th1 and Th2 cells. At the same time, hybrid Th1/2 cells maintained intermediate levels of both GATA-3 protein and Th2 cytokine production (Figure 6B).

During local infection the possibility remains that only a subpopulation of the transferred cells migrates into the footpad and responds to the virus. To challenge hybrid Th1/2 cells and their classic counterparts in a systemic approach, we infected recipients of LCMV-specific Th1, Th2, and hybrid Th1/2 cells intravenously with LCMV. On d 9, at the peak of the effector T cell response, T-bet expression by hybrid Th1/2 cells was Th1-like while GATA-3 expression was intermediate (unpublished data), as seen after local infection (Figure 6B), a behavior mirroring the plasticity of classic Th2 cells [12]. Extending these findings to the memory phase (d 71, i.e., long after viral clearance, which usually occurs within a week), we found that even months after systemic infection, GATA-3 expression and production of IL-4 and IL-13 were maintained in hybrid Th1/2 memory cells at levels in between those of the challenged Th1 and Th2 cells. At the same time, both T-bet and IFN-γ expression of the challenged Th2 and Th1/2 cells was similar to that of Th1 cells (Figure 6C).

Taken together, the Th1/2 phenotype could be quantitatively modulated towards the Th1 or Th2 directions. In this respect, the hybrid cells showed a similar degree of plasticity as their classic Th2 counterparts, which can also acquire additional Th1 cell properties upon LCMV infection [12],[22]. However, the hybrid character as such prevailed even upon vigorous reprogramming attempts, indicating an intriguing stability of a promiscuous differentiation state. This finding is surprising because observations in the stem cell field would suggest such a differentiation state to be only metastable [60],[61]. Thus, with regard to both stability and plasticity, hybrid Th1/2 cells behaved like classic Th cell lineages, indicating that both opposing Th1 and Th2 differentiation programs had been firmly established in naive precursors upon their integration of adverse differentiation signals.

### Balance of Opposing Differentiation Programs in Hybrid Th1/2 Cells Translates into Attenuated Immunopathology

**Figure 7:**
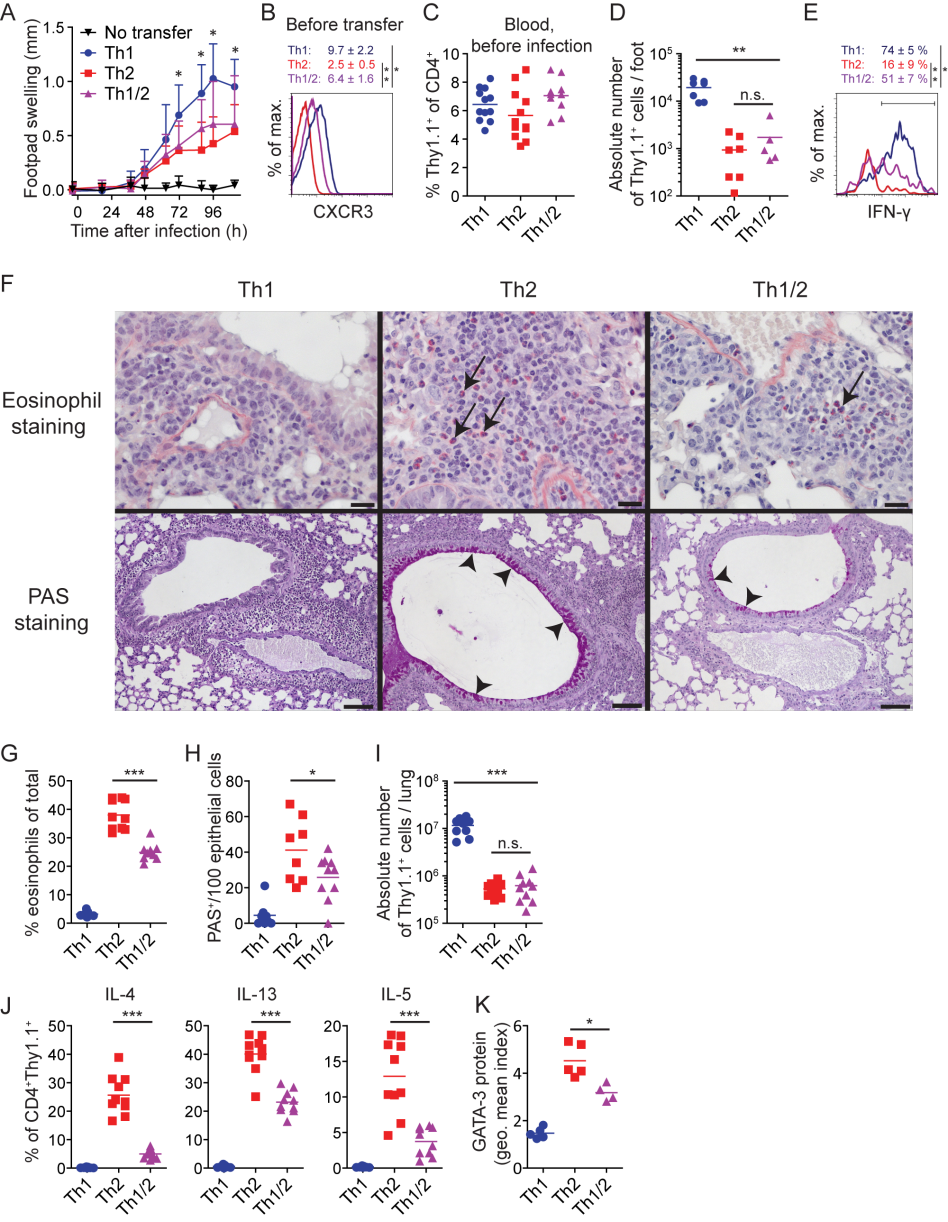
Balance of opposing differentiation programs in hybrid Th1/2 cells translates into attenuated immunopathology. FACS-sorted naive LCMV-TCR^tg^ CD4^+^Thy1.1^+^ T cells were cultured for 5 d with APCs and GP_64–80_ peptide to generate Th1, Th2, or hybrid Th1/2 cells. (A–E) 5×10^6^ cells were transferred into WT mice, followed by subcutaneous LCMV infection in one footpad and sham infection with medium of the other. (A) Footpad swelling was measured in a kinetic fashion as the difference in thickness between the infected and the sham-treated foot (*n* = 10–12 mice/group). Pooled data + SD from two independent experiments are shown. (B) CXCR3 expression was determined before transfer. Representative histogram and geometric means ± SD from four independent experiments are depicted. (C) Frequencies of Thy1.1^+^ donor cells in blood one day before viral infection are shown (*n* = 10–12 mice/group). (D) Absolute number of CD4^+^Thy1.1^+^ cells in challenged feet during the effector phase is depicted (*n* = 5–7 mice/group). (E) IFN-γ expression by CD4^+^Thy1.1^+^ cells isolated from challenged feet was determined upon PMA/ionomycin restimulation during the effector phase on d 5 after infection. Mean frequencies ± SD (*n* = 3–5 mice/group) are indicated. Data are pooled from (C, D) or representative of (E) two independent experiments. (F–K) 1.5×10^6^ cells were transferred into WT mice. Mice were challenged intranasally with 10 µg GP_64–80_ in PBS on 4 consecutive days and analyzed 2 days later. (F) Histological analyses of the lungs. Eosinophils are stained with Sirius Red, and examples are indicated by arrows (scale bar, 20 µm; upper row). Mucus-producing goblet cells in the bronchial epithelium are identified by PAS staining, and examples are indicated by arrowheads (scale bar, 100 µm; lower row, *n* = 4–5 mice/group). (G) Frequency of Siglec-F^+^CD11c^lo^ eosinophils in the lungs of challenged mice was analyzed by FACS (*n* = 9–10 mice/group). (H) Quantification of PAS^+^/100 epithelial cells by histology is shown (*n* = 4–5 mice/group). (I) Absolute numbers of CD4^+^Thy1.1^+^ from the lungs were measured by FACS (*n* = 9–10 mice/group). (J) Frequencies of cytokine^+^ cells upon restimulation with PMA/ionomycin are depicted (*n* = 9–10 mice/group). (K) Geometric mean indices of GATA-3 expression by CD4^+^Thy1.1^+^ cells without restimulation are shown (*n* = 4–5 mice/group). Data are representative of (F, K) or pooled from (G–J) two independent experiments.

To dissect the functional relevance of hybrid Th1/2 cells, we studied both type-1 and type-2 inflammations. As a model of type-1 inflammation, we adoptively transferred *in vitro*–generated, LCMV-specific Th cells and subjected the recipient mice to a local LCMV infection in the footpad [62]. Th1 cells induced a strong delayed-type hypersensitivity (DTH) response. However, footpad swelling in hybrid Th1/2 cell recipients was significantly reduced and only slightly more severe than in mice that had received Th2 cells (Figure 7A). Hybrid Th1/2 cells expressed intermediate levels of CXCR3 (Figure 7B), which is involved in guiding Th cell migration to the inflamed foot [62]. Although the frequencies of transferred cells before infection were comparable between groups (Figure 7C), milder inflammation was accompanied by reduced T cell influx into the feet (Figure 7D). Moreover, in challenged feet of hybrid Th1/2 cell recipients, we detected lower frequencies of IFN-γ producers compared with Th1 cell recipients (Figure 7E).

To assess the type-2 inflammatory potential, we transferred hybrid Th1/2 cells and used a model of allergic airway inflammation by intranasal peptide challenge [63]. (Note that *H. polygyrus* infection is not suitable as a model here, because it is not accompanied by considerable immunopathology.) Hybrid Th1/2 cells mediated significantly less eosinophil accumulation in the lung than Th2 cells (Figure 7F and G). Moreover, we detected a pronounced reduction in mucus-producing goblet cells compared with Th2 cell recipients (Figure 7F and H). The numbers of transferred cells were similar in lungs of Th2 and hybrid Th1/2 cell recipients (Figure 7I). Milder type-2 inflammation was rather associated with reduced IL-4, IL-13, and IL-5 production (Figure 7J) and lower GATA-3 expression (Figure 7K) by the hybrid Th1/2 cells compared with the Th2 cell compartment.

In summary, combined Th1 and Th2 effector functions were exerted by hybrid Th1/2 cells at an intermediate level. Hence, they supported immune responses characteristic of either lineage, however these responses were attenuated in strength and resulted in milder immunopathology.

## Discussion

Here we report the direct commitment of naive Th cells to a stable hybrid phenotype with combined Th1 and Th2 cell functions. Hybrid Th1/2 cells developed in parallel to classic Th2 cells in immune responses against different parasites where they substantially contributed to the type-2 cytokine-producing effector and memory T cell pool. *In vitro* and *in vivo*, the development of hybrid Th1/2 cells was dependent on IFN-γ signaling. In hybrid Th1/2 cells, both Th1 and Th2 features were manifested at an intermediate level.

How can simultaneous Th1 and Th2 differentiation in individual cells be explained? T-bet is first induced by IFN-γ [2],[3],[64]. We found that IFN-γ–STAT1 signaling in developing hybrid Th1/2 cells was not compromised by IL-4 signals. IL-4 drives enhanced expression of GATA-3 [10]. We observed intermediate GATA-3 protein amounts in hybrid Th1/2 cells, most likely due to negative effects of IL-12–STAT4 signals [25] and T-bet [27] on GATA-3 expression. However, intermediate GATA-3 amounts were sufficient to induce substantial production of IL-4 and IL-13 in the presence of T-bet, consistent with the general compatibility of T-bet with Th2 cytokine production [2],[65]. Furthermore, GATA-3 suppresses the expression of both the IL-12Rβ2 chain [25] and STAT4 [26], thereby limiting Th1 cell differentiation. Indeed, we observed suboptimal but functional IL-12 signaling in differentiating hybrid Th1/2 cells. Thus, decreased IFN-γ induction in hybrid Th1/2 cells may either be the direct result of diminished IL-12 signaling [26] or it may occur indirectly via the induction of lower T-bet protein amounts [66],[67]. Moreover, GATA-3–mediated suppression of Runx3 activity [68] may have decreased IFN-γ production.

In the absence of IFN-γ signals, IL-12 could drive only low-level expression of T-bet and IFN-γ. However, this IL-12 effect was completely abrogated by additional IL-4 signals, pointing to their dominance over IL-12 signaling [4],[69]. We found that IFN-γ is capable of breaking the IL-4 dominance, resulting in T-bet and IFN-γ induction in parallel to GATA-3 and IL-4 induction. This finding may be explained at least in part by a role of IFN-γ in sustaining the expression of the IL-12Rβ2 chain [70]. Importantly, in the presence of IFN-γ the entire population of differentiating hybrid cells could perceive IL-12–STAT4 signaling despite concurrent IL-4 signals. Yet IL-12 was not required for hybrid Th1/2 cell development during *H. polygyrus* infection and only partially contributed to T-bet and IFN-γ induction in the presence of IL-4 *in vitro*, suggesting a considerable role of IFN-γ to drive T-bet induction directly [2],[3]. Interestingly, the amounts of STAT1 protein in differentiating hybrid Th1/2 cells were comparable to those in Th1 cells, whereas the amounts of STAT4 protein were comparable to those in Th2 cells. Our results confirm STAT1's relevance for Th1 cell differentiation [2],[71] and show further that STAT1 signaling is most critical when STAT4 signals become limited as by GATA-3–mediated repression. Thus, while the IL-12–STAT4 pathway was subjected to inhibition by GATA-3, it appears to be the IFN-γ–STAT1 pathway that imperviously allowed T-bet induction to occur even in the presence of IL-4 signals. Taken together, in developing hybrid Th1/2 cells mutual repression mechanisms were in place but still allowed the simultaneous induction of the Th1 and Th2 programs.

The co-expression of T-bet and GATA-3 was stably maintained in hybrid Th1/2 memory cells for months. Inhibition of GATA-3 function by T-bet through protein–protein interaction [72] could not impair stable GATA-3 expression, suggesting that GATA-3 autoactivation [24] was largely resistant to such a kind of repression. However, this mechanism may have contributed to reduced GATA-3 expression and decreased per-cell amounts and/or frequencies of Th2 cytokines in hybrid Th1/2 effector cells *in vivo*. Upon LCMV infection, providing very potent Th1-favoring stimuli [12],[22], hybrid Th1/2 memory cells maintained GATA-3 and Th2 cytokine production at intermediate levels while they acquired Th1-like T-bet and IFN-γ expression, reflecting both plasticity and stability of hybrid Th1/2 cells.

Hybrid Th1/2 cells were bifunctional and supported type-1 and type-2 immune responses. However, both were reduced in strength and thus caused milder immunopathology in the type-1 and type-2 inflammation models used here. At least two mechanisms could account for this reduced inflammatory potential. First, DTH responses require Th cells to migrate to inflamed effector sites [73]. After footpad infection with LCMV, CXCR3 ligands are locally expressed [62]. Hence, compared with Th1 cells, reduced influx of hybrid Th1/2 cells into LCMV-infected footpads may have resulted from decreased levels of CXCR3 protein expression. Second, low frequencies of IFN-γ producers among the recruited hybrid Th1/2 cells may have led to low local amounts of IFN-γ and thus to a mild DTH response [74].

In Th2 cell-driven murine asthma models, mucus production and eosinophilia depend on IL-13 and IL-5 [75]. Indeed, hybrid Th1/2 cells from the lungs contained fewer IL-13 and IL-5 producers than Th2 cells, associated with milder eosinophilia and reduced mucus production. Taken together, the intermediate manifestations of Th1 and Th2 effector features were associated with reduced immunopathology mediated by hybrid Th1/2 cells compared with their classic counterparts. Mechanistically, the hybrid phenotype may translate into differential migratory behavior and/or distinct expression patterns of effector molecules at the site of inflammation.

Albeit the expression of the signature cytokines of either lineage was negatively modulated in hybrid Th1/2 cells *in vivo* compared with their classic counterparts, this reciprocal regulation was quantitative rather than qualitative. Thus, even though the hybrid cells expressed IFN-γ and IL-4 in reduced amounts at the per-cell level or in reduced frequencies at the population level, the probability that a given individual IFN-γ–producing cell in the hybrid population also produced IL-4 was the same as the probability that an IFN-γ–negative cell in this population produced IL-4. During a given stimulation of differentiated Th cell populations, cytokine production is generally heterogeneous [16],[17]. Different cytokines can be expressed independently in single cells such that for instance not all IFN-γ–producing Th1 cells also produce IL-2 [17],[76],[77]. Even though it was reported that IFN-γ and type-2 cytokine production in Th cell populations from diverse settings occurs from subpopulations that tend to segregate [17],[76]–[78], a detailed statistical analysis revealed that Th1 and Th2 cytokine expression at the single-cell level often represents statistically independent events [46],[52]. However, since in those studies both the differentiation history and the stability of IFN-γ/IL-4 co-producing cells were unclear, this phenomenon was rather interpreted as incomplete lineage commitment [17],[32],[46],[78]. In contrast, we have here identified Th1/2 cells as a distinct population that homogeneously and stably co-expressed the Th1 and Th2 hallmark transcription factors T-bet and GATA-3. Thus, the fact that not all cells in the hybrid population co-produced IFN-γ and IL-4 does not imply that these cells were not bona fide hybrid Th1/2 cells. The independent decision of restimulated hybrid Th1/2 cells to produce IFN-γ and/or IL-4 rather reflects the probabilistic cytokine expression behavior of classic Th1 and Th2 cell populations. Hence, it indicates that the Th1 and Th2 differentiation programs interact rather weakly and are long-term compatible with each other in individual cells. Besides a stochastic element, molecular events such as the nuclear translocation of NFATc2 or the limited availability of other required transcription factors have been reported to affect the frequency of cytokine production by Th cells and other cell types [79],[80], suggesting that cell-to-cell differences in strength and processing of T cell receptor signals contribute to the heterogeneity of cytokine production observed at the population level. In addition, it is possible that as yet unidentified regulatory factors that may be preferentially expressed in hybrid Th1/2 cells participate in their modulated capacity to produce Th1 and Th2 effector cytokines.

We and others have reported previously that established Th cell subsets can acquire features of other lineages by reprogramming, resulting in hybrid differentiation phenotypes [35]–[37]. Virus-induced type-I and type-II interferon and IL-12 signals could reprogram adoptively transferred fully differentiated Th2 cells to adopt a GATA-3^+^T-bet^+^ phenotype [12]. This virus-triggered T-bet induction was essential because T-bet–deficient Th2 cells fatally interfered with a protective antiviral CD8 T cell response. In the present study, we found that hybrid Th1/2 cells develop naturally *in vivo* in response to parasites. We show that naive Th cells can directly commit to stable T-bet and GATA-3 co-expression by simultaneous initiation of the Th1 and Th2 differentiation programs rather than by reprogramming. For this, IFN-γ was required to overcome the dominance of IL-4 over IL-12 signaling. Importantly, for the first time we characterized the functional properties of a T-bet^+^GATA-3^+^ cell subset and found that the quantitative modulations of both Th1 and Th2 cell functions in hybrid Th1/2 cells translated into reduced immunopathology in different inflammation models.

It has been reported that under some circumstances two lineage-specifying transcription factors may be co-expressed in Th cells during primary immune responses. In these studies, the observed co-expression reflected the signaling of an inductive cytokine that is shared by two lineages [42]–[44],[81]. TGF-β1 induces the Treg cell lineage [82] but is also implicated in the development of Th17 cells [83]. Consistently, TGF-β1 can induce the co-expression of FoxP3 and RORγt, the lineage-defining transcription factors of Treg and Th17 cells, respectively [42]. IL-23 is a key cytokine required for expansion and terminal differentiation of Th17 cells [83],[84] but also induces T-bet [85]. Thus, a subset of pathogenic Th17 cells that co-expressed T-bet and RORγt was dependent on IL-23 signals [43],[44]. Finally, IL-12 not only induces T-bet [9] but also Bcl-6 [81], the lineage-defining transcription factor of Tfh cells [86], leading to the transient expression of Bcl-6 in developing Th1 cells. Some of these reports suggest a role of functional antagonism to resolve the transient hybrid phenotype in favor of a stable classic lineage [42],[81]. In contrast to these findings, we describe here how the processing of opposing developmental cues by pluripotent naive precursors leads to a stable hybrid differentiation phenotype where balanced functional antagonism serves to self-limit the inflammatory potential of Th cells.

The present finding of the direct differentiation of naive Th cells into a hybrid T-bet^+^GATA-3^+^ Th1/2 phenotype is also of interest from a broader systems-biology perspective. One reason for the wide acceptance of the idea of mutually exclusive Th1 and Th2 phenotypes might be that mutual repression in a gene regulatory network, such as the one governing T-bet and GATA-3 expression, can generate two distinct “basins of attraction”, corresponding to two different cell types. Indeed, cell fate “branch points” associated with this concept have recently attracted attention in stem cell biology [60],[61]. However, the effect of mutual repression depends on quantitative parameters of the underlying interactions. Already quite simple models of GATA-3 and T-bet expression predict that both mutual exclusion and co-expression might occur, depending on the strength of repression, where co-expression is favored by weak interactions [87]. A quantitative model of Th cell differentiation will require a detailed mechanistic understanding of both induction and maintenance of the differentiated phenotypes. Our data point to a model with weak antagonistic interactions between Th1- and Th2-inducing pathways. The T-bet and GATA-3 expression levels attained in the induction phase are then “remembered” in the memory phase of the immune response and remain only partially susceptible to later reprogramming.

It is a common theme in developmental biology that progenitor cells destined to give rise to either of two cell lineages co-express the master transcription factors of both [60],[61]. Lineage commitment is then achieved by the loss of expression of one factor in favor of stable elevated expression of the other. We cannot formally exclude that also the differentiation of Th1 and Th2 cells passes through a transient hybrid stage where T-bet and GATA-3 are co-expressed, although we did not detect it in detailed kinetic analyses. In general, Th cell differentiation appears to be rather based on the *de novo* induction of master transcription factors in naive progenitor cells that do not co-express all the transcription factors that define the different lineages. Moreover, the hybrid Th1/2 state is more firmly established than it would be expected from a metastable system since even vigorous reprogramming attempts rather induced quantitative modulations of the hybrid phenotype but not its loss. Transcriptional autoactivation has been suggested to stabilize the expression of the Th1 and Th2 lineage-specifying master transcription factors T-bet [2],[23] and GATA-3 [24],[88], respectively. Weak mutually inhibitory interactions between the pathways inducing them would then modulate the expression levels without abolishing co-expression [87],[89].

In summary, we conclude that mechanisms of reciprocal inhibition do not necessarily make lineage commitment of naive Th cells an exclusive choice between alternative cell fates. Instead, the integration of adverse polarizing signals by a differentiating cell can result in the acquisition of stably balanced functional properties endowed by two opposing transcriptional programs. We suggest that mutual inhibition may rather form the basis of this cell-intrinsic balance—for example, by leading to quantitative differences in inductive signal strength [90], which result in a hybrid phenotype with dual functions where each is exerted at an intermediate level.

Yet threshold levels may exist beyond which classic lineages can stabilize and initiation of opposing programs is prevented. Indeed, in responses against parasites we observed the parallel development of T cells with a classic Th2 and a hybrid Th1/2 phenotype. GATA-3^+^ populations were not distributed over a T-bet continuum but instead emerged as distinct populations that were either T-bet^−^GATA-3^+^ or T-bet^+^GATA-3^+^, indicating that a certain threshold of T-bet expression may have to be reached in order to establish the hybrid Th1/2 phenotype.

In functional terms, the acquisition and maintenance of a hybrid Th cell phenotype could contribute to the prevention of excessive immunopathology as an effector cell-intrinsic mechanism. We propose a regulatory mechanism that is not based on immunosuppression but on the quantitative modulation of immune cell functions achieved by the balance of two antagonistic differentiation programs. We note that direct differentiation of naive CD4^+^ T cells into a mixed Th1/Th2 phenotype i*n vitro* was also observed concurrently by two other groups, using different experimental approaches [98],[99].

## Materials and Methods

### Mice

LCMV–TCR^tg^ (SMARTA1) [91] Thy1.1^+^ mice expressing a TCR specific for the LCMV epitope GP_61–80_ on C57BL/6 background, as well as WT C57BL/6 and WT Balb/c mice or *Ifngr1^−/−^* mice [92] were used as organ donors for the isolation of splenocytes and lymph node cells. Normal untreated C57BL/6J mice were used as recipients in adoptive cell transfer experiments. *Ifngr1^−/−^* mice, *Ifnar1^−/−^* mice [92], *Il12p40^−/−^* mice [93], *Il4^−^* ^/*−*^ mice [94], and *Il4ra^−^* ^/*−*^ mice [95] were all on C57BL/6 background. These mice as well as WT C57BL/6 and WT Balb/c mice were used in infection and immunization experiments, as indicated in the figure legends. Mice were bred under specific pathogen-free conditions at the Charité, Berlin, or at the University of Zurich, Switzerland. All animal experiments were performed in accordance with the German and the Swiss law for animal protection with permission from the local veterinary offices.

### Primary T Cell Cultures

Naive CD4^+^CD62L^hi^CD44^lo^CD25*^−^*CXCR3*^−^*Gr-1*^−^* T cells were sorted from pooled spleen and lymph node cells by FACS to a purity of >99.5%. T cells were cultured in RPMI 1640+GlutaMax-I supplemented with 10% (v/v) FCS (Gibco), penicillin (100 U/ml; Gibco), streptomycin (100 µg/ml; Gibco), and ß-mercaptoethanol (50 ng/ml; Sigma). Cultures were prepared either in the presence of APCs and 0.5 µg/ml LCMV–GP_64–80_, (R. Volkmer, Institute for Med. Immunology, Charité) or by stimulation with plate-bound 2.5 µg/ml anti-CD3ε (145-2C11) and 3 µg/ml anti-CD28 (37.51, both from BD Biosciences). For Th1 differentiation, 10 ng/ml IFN-γ, 10 ng/ml IL-12 (R&D Systems), and 10 µg/ml anti–IL-4 (11B11) were added. For Th2 differentiation, 30 ng/ml IL-4 (R&D Systems), 10 µg/ml anti–IL-12 (C17.8), and 10 µg/ml anti–IFN-γ (AN18.17.24) were added. Hybrid Th1/2 cells were cultured with 10 ng/ml IFN-γ, 10 ng/ml IL-12, and 30 ng/ml IL-4. For Th17 differentiation, 20 ng/ml IL-6, 1 ng/ml TGF-β, 10 ng/ml IL-23 (all from R&D Systems), 10 µg/ml anti–IFN-γ, and 10 µg/ml anti–IL-4 were added. For some experiments, cells were cultured under neutral conditions with anti–IL-12, anti–IFN-γ, and anti–IL-4. For *in vitro* Th1 reprogramming experiments, IFN-α and IFN-β (PBL InterferonSource) were used at 1,000 U/ml. Cell cultures were split on d 2 or d 3 and analyzed on d 5. For a second round of stimulation, cells were reactivated on d 5 with fresh Thy1.2-depleted C57BL/6 splenocytes plus GP_64–80_ peptide, 5 ng/ml IL-2 (R&D Systems), polarizing cytokines, and cytokine-blocking antibodies as above.

### Viruses and Parasites

The LCMV-Armstrong and LCMV-WE strains were propagated on BHK or L929 cells, respectively, and virus stocks were titrated by standard immunofocus assays. *H. polygyrus* was maintained by serial passage in C57BL/6 mice. *Biomphalaria glabrata* snails infected with *S. mansoni* were maintained at the Institute of Molecular Parasitology (Berlin, Germany). *S. mansoni* eggs were recovered from the livers of infected hamsters, washed, and frozen.

### Adoptive T Cell Transfer

Mice were injected intravenously with differentiated LCMV-specific CD4^+^Thy1.1^+^ cells in 500 µl PBS. In cases where the recipients were infected with LCMV later on, 1–5×10^6^ cells were transferred to yield a seeding of approx. 1–5×10^5^ cells per mouse [96]. In some experiments where the recipients were left uninfected, 1×10^7^ cells were transferred to yield a seeding of approx. 1×10^6^ cells per mouse.

### Infections and Immunizations

#### LCMV

Preceding systemic LCMV infection, 1×10^6^ cells were transferred per mouse. One week later, mice were infected intravenously with 200 plaque-forming units (PFU) of LCMV-WE in 200 µl medium.

Prior to local LCMV infection, 5×10^6^cells were transferred per mouse. One week later, mice were infected with 500 PFU of LCMV-Armstrong in 30 µl medium into the left footpad. Equal volumes of medium were injected into the right footpad as a control. Footpad swelling was measured using a caliper and calculated as the difference in footpad thickness of infected versus control feet. Mice were sacrificed during the effector phase (d 5 to d 8 after infection).

#### *H. polygyrus*

Mice were infected with 200 L3 larvae via oral gavage and analyzed between d 14 and d 21 (peak of infection). For some experiments, mice were treated orally with 2.5 mg Pyrantel pamoate (Sigma) in 150 µl water 3 wk after primary infection. Abrogation of infection was verified using MacMaster chambers confirming absence of parasite eggs in feces.

#### *S. mansoni*

Female Balb/c mice were infected subcutaneously with 90 infective stage cercariae derived from snails and analyzed 8 wk after infection.

#### *S. mansoni* eggs

WT Balb/c mice were immunized twice with 2,000 *S. mansoni* eggs in PBS i.p. on d 0 and d 30. One month later, mice received 2,000 eggs in PBS i.v. and were sacrificed 8 d later.

### Airway Inflammation Model

1.5×10^6^ cells were transferred into WT mice and rested *in vivo* for 1 wk. Mice were challenged by intranasal inoculation with 10 µg GP_64–80_ in PBS on 4 consecutive days. On d 2 after the last challenge, mice were analyzed.

### Cell Isolation and Flow Cytometry

Viable T cells from primary cell cultures were purified using Histopaque 1083 (Sigma-Aldrich) and density centrifugation (400 *g* at 25°C for 20 min). Single-cell suspensions of spleen and lymph nodes were prepared by mechanical disruption.

For cell isolation from lungs, livers, and footpads, mechanical disruption was followed by digestion with Collagenase D (0.1 U/ml, Roche) for 30 min at 37°C. Then, lymphocytes were isolated using Histopaque 1083 and density centrifugation as described above.

Intestines were digested with Collagenase D (0.1 U/ml, Roche) and Collagenase VIII (212 U/ml, Sigma-Aldrich) after removal of Peyer's patches and intensive washing in RPMI 1640+GlutaMax-I. Then, lymphocytes were isolated using Percoll (Biochrom) and density gradient centrifugation (400 *g* at 25°C for 20 min).

Adoptively transferred T cells were identified by an antibody to Thy1.1 (OX-7, eBioscience). To prevent unspecific binding of antibodies, all samples were co-incubated with 10 µg/ml anti-FcγRII/III (2.4G2; ATCC) and 2.5 µg/ml purified rat IgG (Jackson Immuno Research). When indicated, cells were stained with antibodies to CXCR3 (CXCR3-173), CD11c (N418), Gr-1 (RB6-8C5, all from eBioscience), IL-18Rα (BG/IL18RA), CCR4 (2G12), CCR6 (29-2L17, all from Biolegend), Siglec F (E50-2440), and CD25 (7D4, both from BD). Samples were sorted on a FACS Aria II, acquired on a FACS Canto II (Becton Dickinson), and analyzed with FlowJo (Tree Star). Dead cells and doublets were excluded by a combination of forward scatter height and width gating and the usage of propidium iodide or a LIVE/DEAD fixable dye (Invitrogen).

### Intracellular Cytokine Staining

For intracellular analysis of cytokines, cells were restimulated with either GP_64–80_ (1 µg/ml) or PMA (5 ng/ml) and ionomycin (500 ng/ml) for 4 h with addition of brefeldin A (5 µg/ml; all from Sigma-Aldrich) after 30 min. Antigen-specific restimulation of cells from *H. polygyrus*–infected mice was performed using 100 µg/ml adult worm lysate for 6 h with addition of brefeldin A after 2 h, identifying specific cells using an antibody to CD40L (MR1, MiltenyiBiotec). Following restimulation, cells were fixed in 2% formaldehyde (Merck). Intracellular staining was performed in PBS/0.2% BSA containing 0.05% saponin (Sigma-Aldrich) for permeabilization. Samples were stained with antibodies to CD4 (RM4–5), Thy1.1 (OX-7), IFN-γ (XMG1.2), IL-4 (11B11), IL-5 (TRFK5), IL-10 (JES5-16E3), and IL-13 (38213.11, eBio13A).

### STAT Stainings

STAT protein amounts and phosphorylation of STAT proteins were analyzed using BD Phosflow buffers according to the manufacturer's instructions (BD Biosciences). Briefly, cells were fixed with prewarmed 1× BD Phosflow Lyse/Fix Buffer for 10 min at 37°C. Cells were permeabilized with ice-cold BD Phosflow Perm Buffer III for 30 min on ice. Then, cells were stained for 30 min with anti-CD4 and either PE-conjugated anti-pSTAT1 (4a) and Alexa-647–conjugated anti-STAT1 (1/Stat1) or PE-conjugated anti-pSTAT6 (pY641) and Alexa-647–conjugated anti-STAT6 (23/STAT6) or PE-conjugated anti-pSTAT4 (38/p-Stat4; all from BD Biosciences) and polyclonal rabbit anti-STAT4 (Zymed). Secondary antibody (Cy5-conjugated donkey anti-rabbit; Jackson Immunoresearch) was added at a final concentration of 0.2 µg/ml. Cells were washed and analyzed by FACS. For analysis of phosphorylated STAT proteins, the geometric mean index was calculated as the geometric mean of the analyzed population divided by the geometric mean of cells cultured under neutral conditions. For analysis of total STAT proteins, the geometric mean index was calculated as the geometric mean of the analyzed population divided by the geometric mean of the respective isotype control-stained population.

### T-bet and GATA-3 staining

T-bet and GATA-3 protein amounts were analyzed using FoxP3 staining buffer set (eBioscience) according to the manufacturer's instructions. Briefly, cells were stained with anti-CD4 (RM4–5), anti-CD62L (MEL-14), anti-CD44 (IM7), and anti-Thy1.1 (OX-7) followed by fixation with 1× Fixation/Permeabilization buffer and intracellular staining with PE-conjugated anti–T-bet (4B10) and Alexa-647–conjugated anti–GATA-3 (TWAJ, both from eBioscience) in 1× permeabilization buffer. Cells were washed in 1× permeabilization buffer and analyzed by FACS.

Geometric mean indices were calculated as the geometric mean of stained cells divided by the geometric mean of isotype control-stained cells. For *ex vivo* analyses, endogenous CD4^+^CD62L^hi^CD44^lo^ naive cells were used as an additional internal control such that geometric mean indices of the analyzed populations were normalized to the geometric mean index of endogenous naive cells.

### Blimp-1 and Bcl-6 Stainings

Blimp-1 and Bcl-6 protein amounts were analyzed following the protocol described for T-bet and GATA-3 stainings, using PE-conjugated anti–Blimp-1 (C-21, Santa Cruz) or anti–Bcl-6 (K112-91, BD) antibodies. Geometric mean indices were calculated as the geometric mean of Th1, Th2, or hybrid Th1/2 cells divided by the geometric mean of control Tfh cells from spleens of WT C57BL/6 mice, identified by the co-expression of CXCR5 (2G8, BD) and PD-1 (J43, eBioscience).

### Histology

One lobe of each individual lung was fixed in 4% formalin at room temperature for 24 h. After embedding in paraffin, sections were stained with either periodic acid-Schiff (PAS) using standard protocols for goblet cell quantification or with hematoxylin/Sirius red for visualization of eosinophils. Briefly, for eosinophil staining, deparaffinized sections were stained with hemalum for 1 min followed by staining with Sirius red (Direct Red80 from Sigma) for 2 h according to published protocols [97].

A Zeiss Axio Imager Z1 microscope was used and pictures were taken with a Zeiss Axio Cam MRc camera and analyzed with Axio vision software. An ocular ×10 and objective ×10 (PAS staining) or ×40 (Sirius red staining) were used, resulting in a 100-fold or 400-fold magnification, respectively. For PAS staining analysis, the number of PAS^+^ goblet cells among 100 epithelial cells was counted five times.

### Statistical Analysis

Two groups were compared with two-tailed unpaired Student's *t* test; n.s., not significant; * *p*<0.05, ** *p*<0.01, *** *p*<0.001.

## Supporting Information

Figure S1 **IFN-γ, IL-12, and IL-4 constitute the optimal signal combination for the differentiation of hybrid Th1/2 cells** ***in vitro*** **.** FACS-sorted naive CD4^+^CD62L^hi^CD44^lo^CD25^−^CXCR3^−^Gr1^−^ Th cells from WT C57BL/6 (upper two rows) or *Ifngr1* ^−/−^ (lower two rows) mice were activated with anti-CD3/anti-CD28 in the presence of the indicated cytokines and cytokine-blocking antibodies. T-bet and GATA-3 expression was analyzed on d 5 (first and third row). Inserted numbers indicate geometric mean indices. Cytokine expression was analyzed upon PMA/ionomycin restimulation on d 5 (second and fourth row). Numbers indicate frequencies. Data are representative of two independent experiments.
(TIF)

## Abbreviations

APC: antigen-presenting cell
DTH: delayed-type hypersensitivity
GP: glycoprotein
LCMV: lymphocytic choriomeningitis virus
mLN: mesenteric lymph nodes
PAS: periodic acid-Schiff
