# Supplementary figures and images for: Stable T-bet+GATA-3+ Th1/Th2 Hybrid Cells Arise In Vivo, Can Develop Directly from Naive Precursors, and Limit Immunopathologic Inflammation
Source: PLoS Biol. 2013 Aug 20;11(8):e1001633. doi: 10.1371/journal.pbio.1001633 (PMC3747991; doi:10.1371/journal.pbio.1001633)

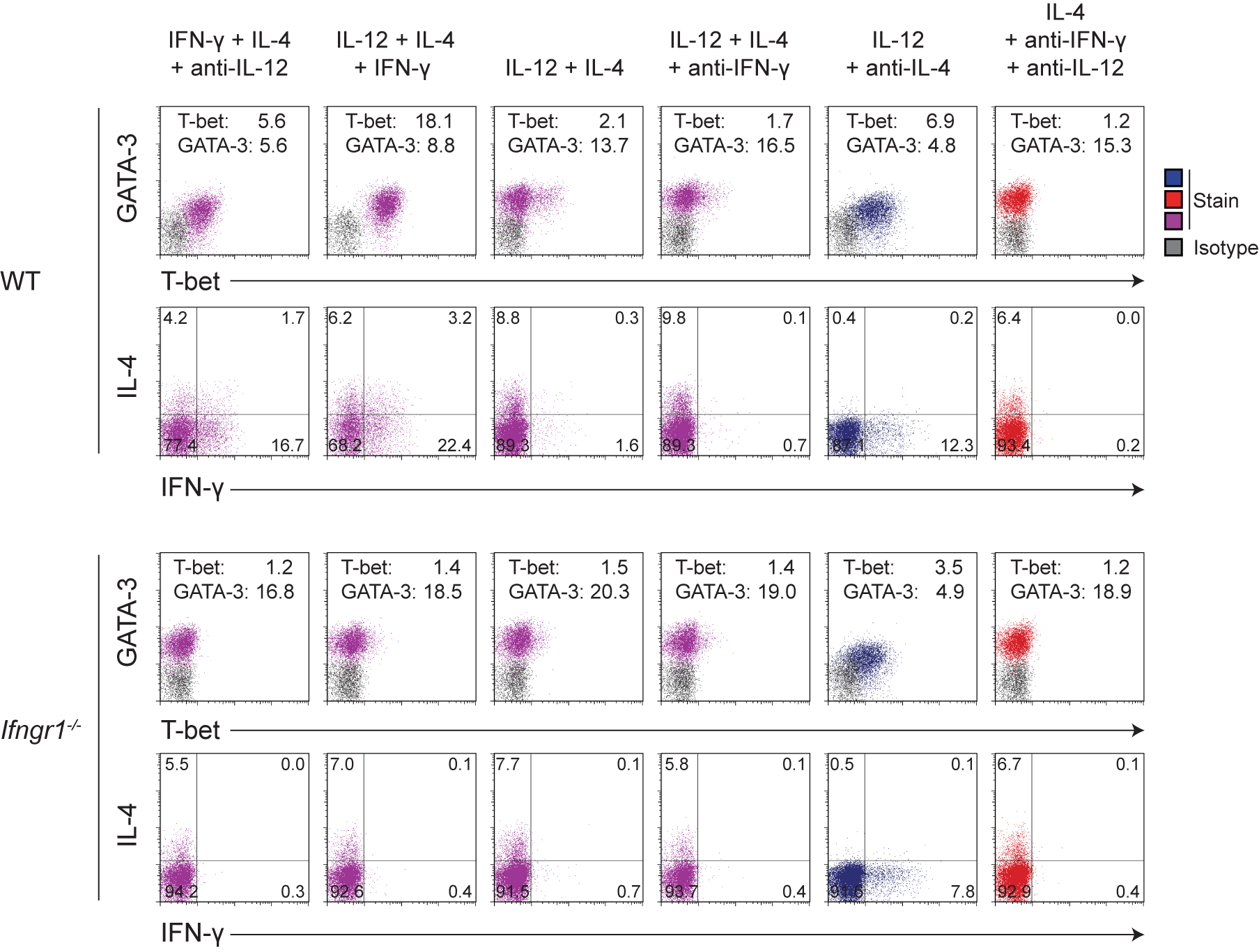

Supplement: Figure S1 — IFN-γ, IL-12, and IL-4 constitute the optimal signal combination for the differentiation of hybrid Th1/2 cells in vitro . FACS-sorted naive CD4+CD62LhiCD44loCD25−CXCR3−Gr1− Th cells from WT C57BL/6 (upper two rows) or Ifngr1 −/− (lower two rows) mice were activated with anti-CD3/anti-CD28 in the presence of the indicated cytokines and cytokine-blocking antibodies. T-bet and GATA-3 expression was analyzed on d 5 (first and third row). Inserted numbers indicate geometric mean indices. Cytokine expression was analyzed upon PMA/ionomycin restimulation on d 5 (second and fourth row). Numbers indicate frequencies. Data are representative of two independent experiments. (TIF) [file pbio.1001633.s001.tif]
